# Innovative Approach in the Management of Displaced Mandibular Fracture in a Four-Year-Old Child: A Case Report

**DOI:** 10.7759/cureus.78038

**Published:** 2025-01-26

**Authors:** Varsha Sharma, Priti Shukla, Shivesh Acharya, Ravinder S Bedi

**Affiliations:** 1 Dentistry, Maharshi Vashishtha Autonomous State Medical College, Rampur, IND; 2 Orthodontics and Dentofacial Orthopedics, All India Institute of Medical Sciences, Raebareli, Raebareli, IND; 3 Pediatric Dentistry, All India Institute of Medical Sciences, Raebareli, Raebareli, IND; 4 Dentistry, All India Institute of Medical Sciences, Raebareli, Raebareli, IND

**Keywords:** cap splint, displaced jaw fracture, mandibular fracture, pediatric fracture, unfavorable fracture of jaw

## Abstract

Craniofacial trauma has been reported to be a major health issue in children. In the pediatric population, the mandible is a more common site than the maxilla. The condyle and parasymphysis are the major sites of mandibular fracture in children. The encountered mandibular fractures are undisplaced due to the high elasticity of the mandible and the less dense condylar neck that resists the displacement of the bone. Management is more directed toward less invasive procedures without manipulating the facial skeleton such that it results in less psychological and physical trauma to the child. The presented case is about the management of a severely displaced, unfavorable fracture of the mandible. Closed reduction by acrylic splints with circum-mandibular wiring is always an ideal treatment choice for mandibular fractures in children. However, in severely displaced mandibular fractures, open reduction and internal fixation (ORIF) remains the best choice. To overcome the downside of the ORIF, a modified cap splint with a horseshoe-shaped wire framework was constructed and named the “Functional Ease Cap Splint.”

## Introduction

Craniofacial fractures have been reported to be less common in children as compared to adults [1]. In the pediatric population, the mandible is a more common site than the maxilla. The condyle and parasymphysis account for the major site of mandibular fracture in children. The incidence of mandibular fractures is rare in preschool children (0.6-1.4%), while it increases in school-going groups and reaches the zenith in adolescents due to an increase in contact sports [2]. The unattended and unsupervised activity of kids is a common factor for condylar fractures in the school-going age group. The encountered mandibular fractures are undisplaced due to the high elasticity of the mandible and the less dense condylar neck that resists the displacement of the bone [3]. The treatment modalities for displaced and undisplaced fractures differ in their management. Several authors have demonstrated techniques like circumferential wiring, cap splints, orthodontic resin, modified bracket techniques, and bone plates for displaced unfavorable fractures, but the preferred treatment of choice is open reduction and immobilization [4]. In the pediatric population, the treatment approach varies for adults due to the presence of permanent tooth buds and growth centers. Management is more directed toward less invasive procedures without manipulating the facial skeleton such that it results in less psychological and physical trauma to the child.

This case reports about the management of a displaced unfavorable fracture of the mandible in which occlusion was disturbed. A modified cap splint with a wire framework was fabricated to enhance the strength of the splint against the muscle pull and fracture displacement.

## Case presentation

A four-year-old healthy boy was reported to the department of dentistry at All India Institute of Medical Sciences, Raebareli, India, with a history of falling from the terrace in the morning on the same day. There was no history of loss of consciousness, vomiting, associated convulsion, or bleeding from the nose and ear.

**Figure 1:**
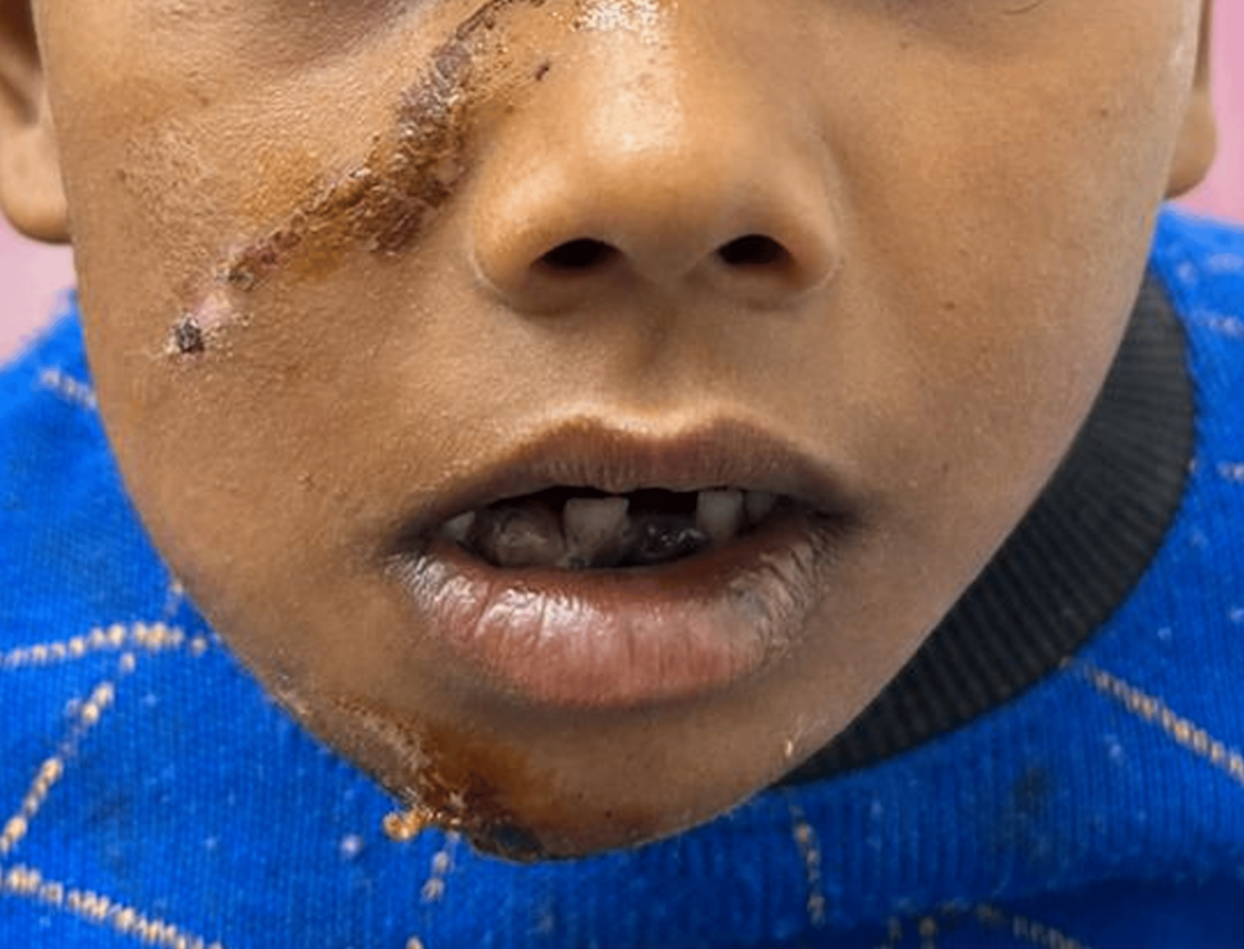
Preoperative extraoral photograph showing facial asymmetry and swelling

Extraoral examination affirmed multiple lacerations all over the face, a deviation of the mandible toward the right, giving an asymmetrical appearance to the face (Figure 1).

**Figure 2:**
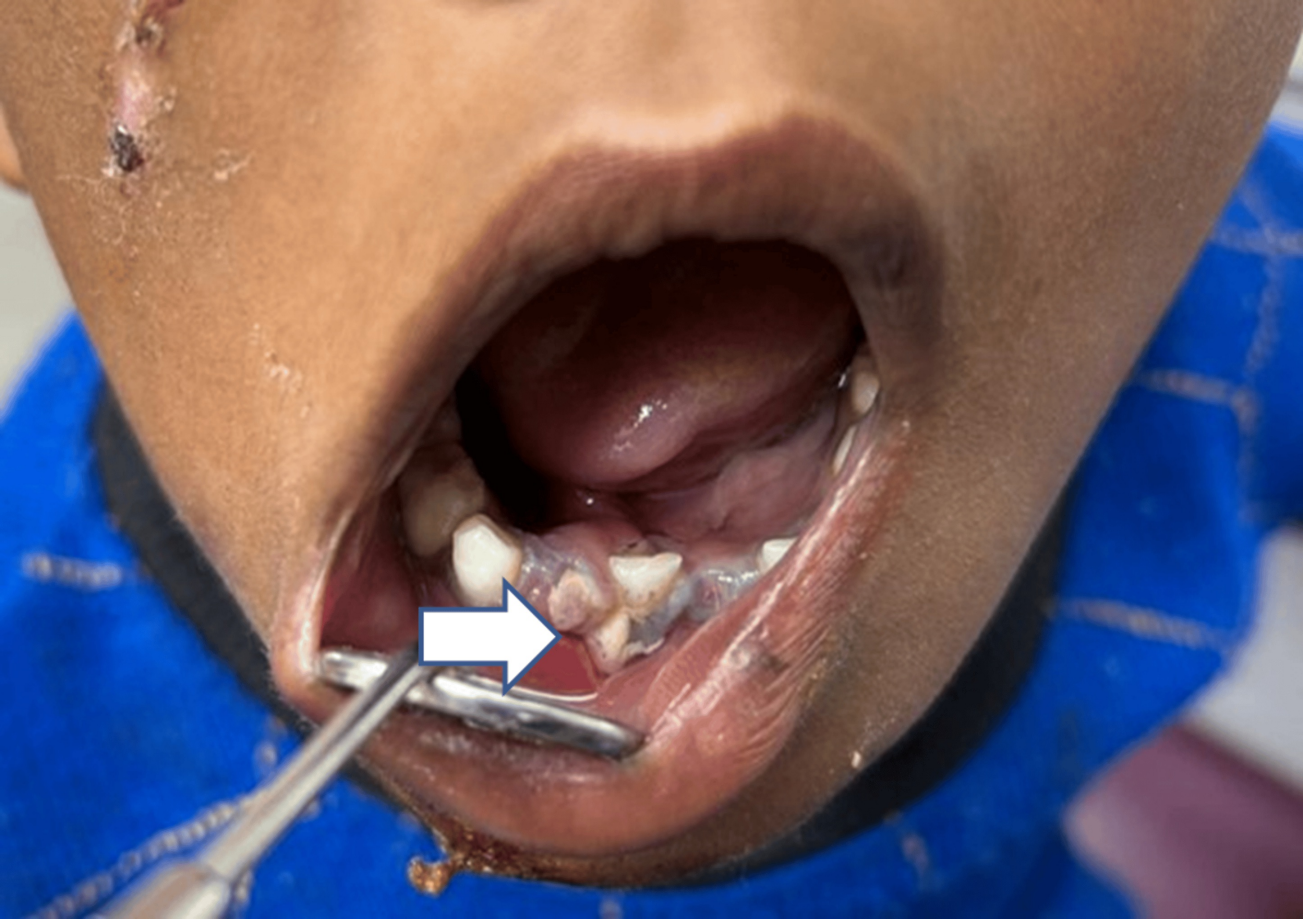
Preoperative intraoral photograph showing step deformity in the 82, 83 region

Intraoral examination affirmed restricted mouth opening with step deformity in respect to 82, 83. The child was in the primary dentition phase (Figure 2).

Radiological examination

**Figure 3:**
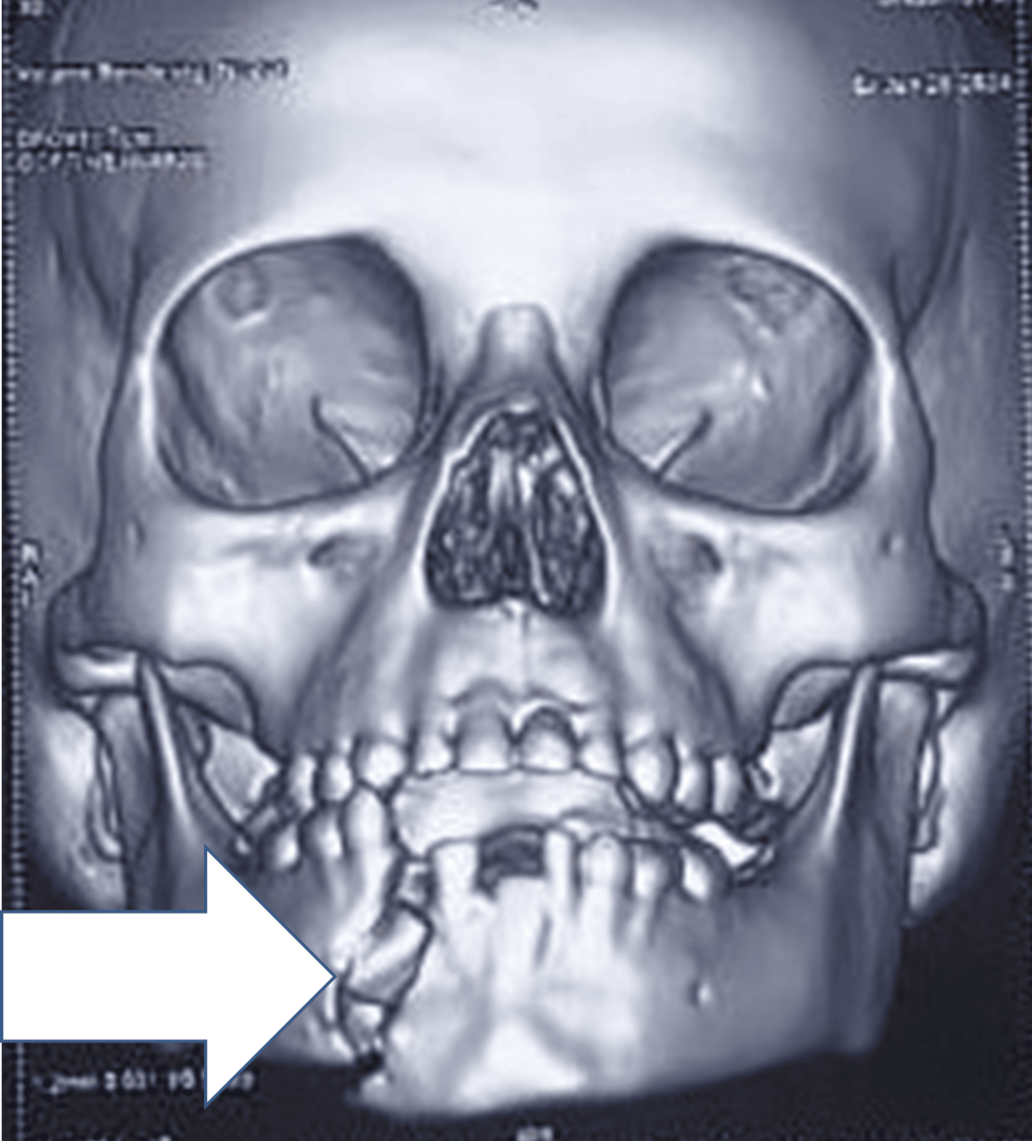
Facial computed tomography (CT) scan with 3D reconstruction illustrating the displaced fracture site

A facial computed tomography (CT) scan with 3D reconstruction revealed a displaced, unfavorable fracture of the right parasymphysis (Figure 3). Occlusion was highly deranged.

Treatment plan

A closed reduction of the mandibular fracture was planned. Weighing the severe displacement nature of the mandible and the child’s age, the conventional cap splint was modified using a horseshoe-shaped wire framework followed by circum-mandibular wiring.

Modified cap splint with horseshoe-shaped wire framework: constructional steps

**Figure 4:**
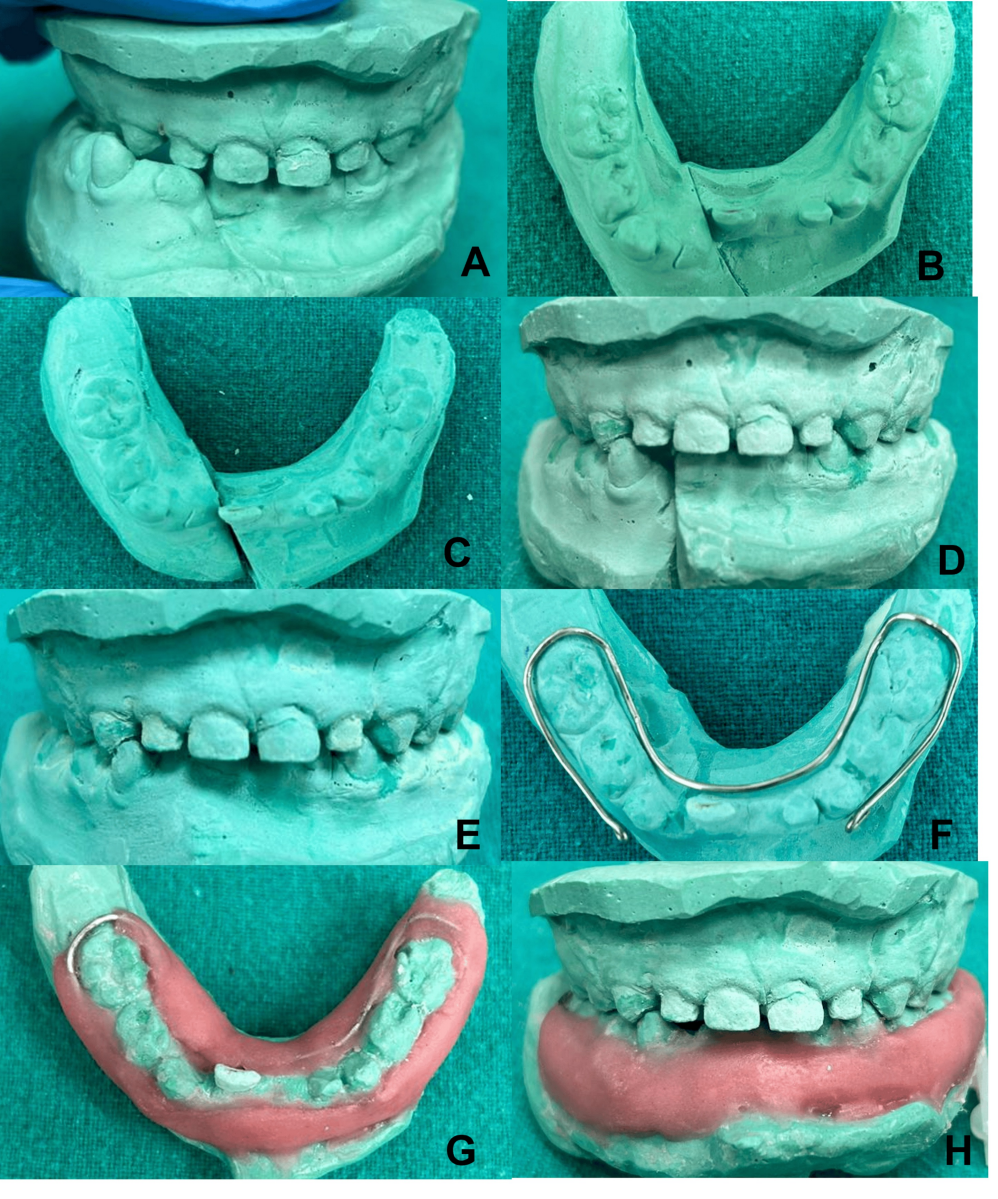
Constructional steps of cap splint A mandibular cast was simulated for the fracture line and was sectioned with the help of a dental plaster saw (A-C). Stabilization of the casts in occlusion (D-E). Horseshoe-shaped framework (F) and construction of final acrylic functional cap splint (G-H).

The steps are as follows: 1) The primary impression of the maxilla and mandible was registered with irreversible hydrocolloid using impression tray size number 0; 2) Impressions were poured with dental stone. A mandibular cast was simulated for the fracture line and was sectioned with the help of a dental plaster saw (Figures 4A-4C); 3) Both the mandibular segments were oriented with a maxillary cast and were stabilized with dental wax (Figures 4D-4E). The maxillary and mandibular cast was articulated using canine guidance; 4) The horseshoe-shaped framework was fabricated using 19-gauge (G) stainless steel wire on the mandibular cast (Figure 4F), and the final acrylic functional cap splint was constructed (Figures 4G-4H).

**Figure 5:**
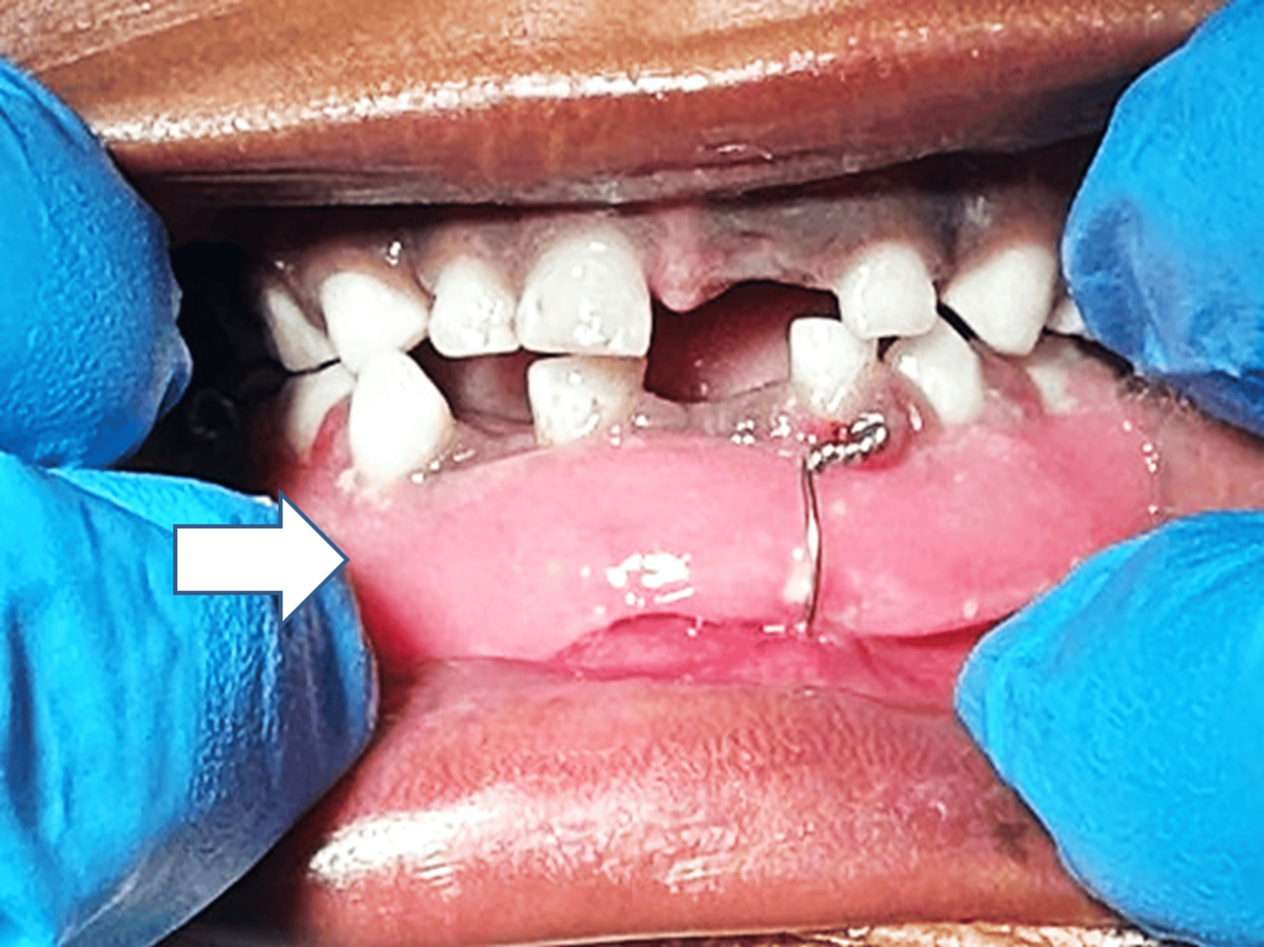
Intraoral photograph: placement of cap splint

**Figure 6:**
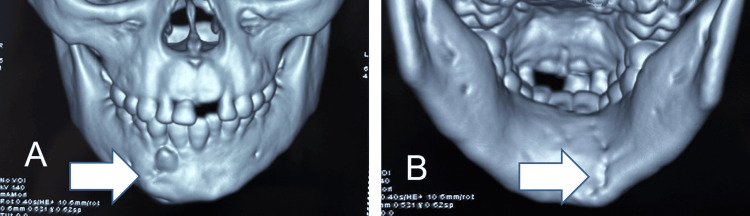
Postoperative CT scan A: frontal view, B: lingual view Arrow depicts the healed fracture site

This procedure was done under general anesthesia to reduce the trauma to the child during fracture reduction (Figure 5). No postoperative complication was experienced. Removal of the splint was done after three weeks. Weekly follow-up was done for the first two months, followed by monthly follow-ups till eight months postoperative. A CT was done at eight months follow-up (Figure 6).

## Discussion

Traumatic oro-dental injuries have been the leading cause of compromised well-being in children, leading to pain, discomfort, and a lasting psychological impact. Such an occurrence affects their quality of life by decreasing their social interaction and increasing their absence from school. Various guidelines have been imposed to safeguard them, but still, it takes a toll when the child grows old.

An important consideration in the pediatric population is their nutritional, fluid, and electrolyte intake and airway maintenance throughout the treatment [5]. Preferably high protein diet is recommended. The time elapsed in anatomic reduction is critical in children as they have high osteogenic potential and revival rates in contrast to adults. As a consequence, it must be quicker, and immobilization is also required for a shorter time duration (two weeks as compared to four weeks for adults) [6,7].

In children with displaced mandibular fractures, intermaxillary fixation (IMF) with arch bars and eyelets is always questionable due to the unstable anchorage system because of the presence of the resorbing primary roots, attrited primary teeth [8], and soft pliable bony architecture [9].

Recently, open reduction with resorbable bone plates has been the treatment of choice for highly displaced mandibular fractures [5,10]. In spite of the fact that open reduction and internal fixation (ORIF) results in primary bony union, speedy healing, and better 3D firmness, its disadvantages prevail over them. Threat analog in the pediatric population with ORIF is an injury to developing permanent tooth germs and crypts, retarding the growth of jaws, plate migration, and sometimes allergic reactions [11]. Therefore, a cap splint with circum-mandibular wiring is mostly the treatment of choice. It allows the child to do all the daily activities with less pain and discomfort.

In the presented case report of a severely displaced mandible, authors pioneered the fabrication of a more robust and sturdier framework to overcome the displacement caused by muscle pull, which is usually seen in conventional cap splints. The horseshoe-shaped framework constructed using 19-gauge (G) stainless steel wire keeps the fractured segments in place even in adverse conditions like acrylic splitting while maintaining the functionality of the splint in the healing phase. The functional nature of this modified cap splint adds additional benefit to the child in meeting his nutritional needs. Thus it was named a “Functional Ease Cap Splint” as it was both easy to construct and sturdy to carry out functional movements.

## Conclusions

Closed reduction by acrylic splints with circum-mandibular wiring is an ideal treatment choice for mandibular fractures in children. But in severely displaced mandibular fractures, ORIF remains the best choice. A modified cap splint with a horseshoe-shaped wire framework should be constructed in isolated fractures of the body of the mandible so that the downsides of ORIF can be decreased in such cases.
